# Supplementary material for: Changes in Alprazolam Metabolism by CYP3A43 Mutants
Source: Biomedicines. 2022 Nov 23;10(12):3022. doi: 10.3390/biomedicines10123022 (PMC9775082; doi:10.3390/biomedicines10123022)
Supplement: Supplementary file 1 [file biomedicines-10-03022-s001.zip › biomedicines-1971697-supplementary.pdf]

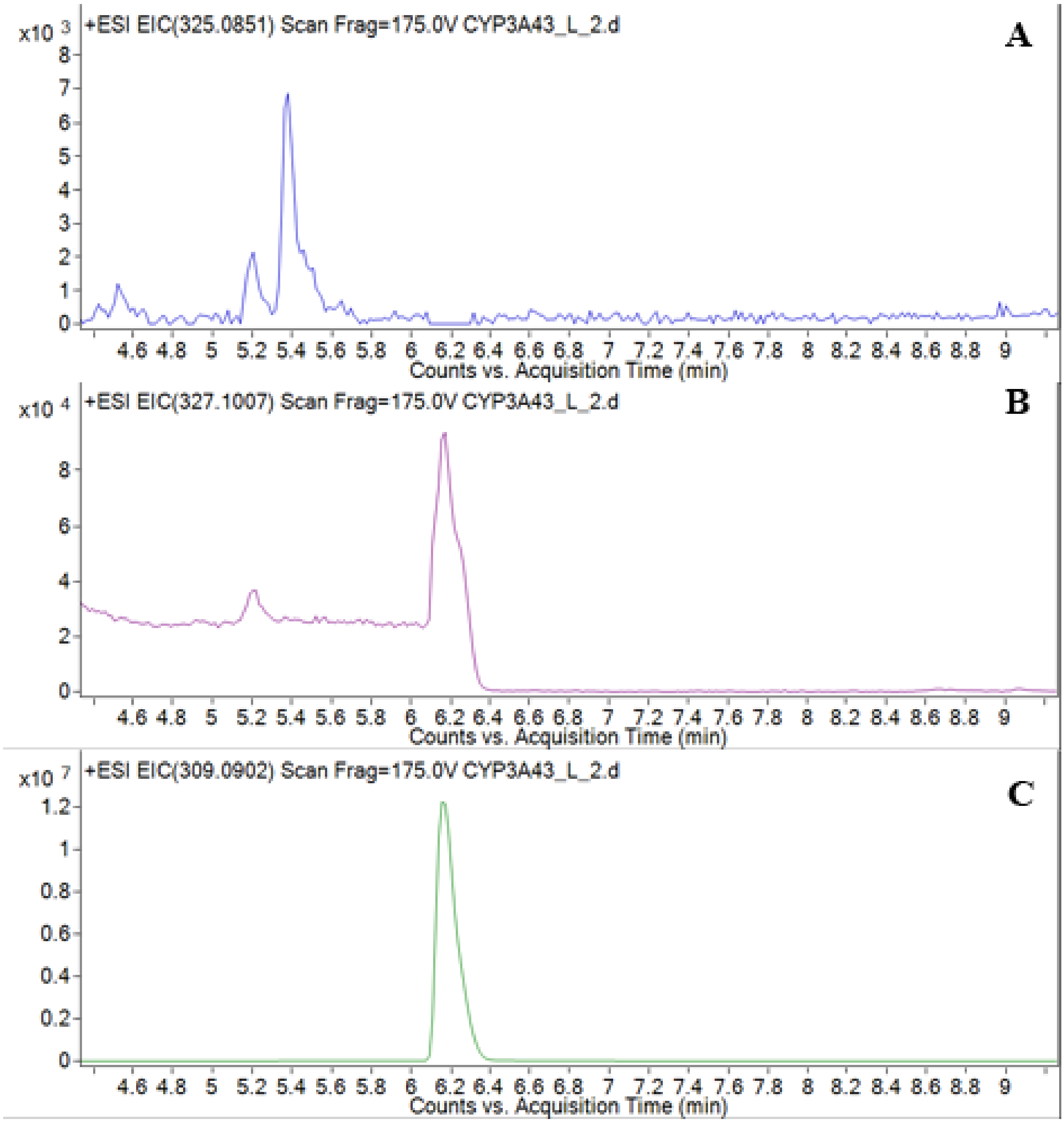


**Figure S1** EIC spectra of CYP3A43 L293P biotransformated alprazolam samples *m/z* 325.0851 (($[C_{17}H_{13}ClN_4O+H]^+$, **A**), *m/z* 327.1007 ($[C_{17}H_{15}ClN_4O+H]^+$, **B**) and *m/z* 309.0902 ($[C_{17}H_{13}ClN_4+H]^+$, **C**).

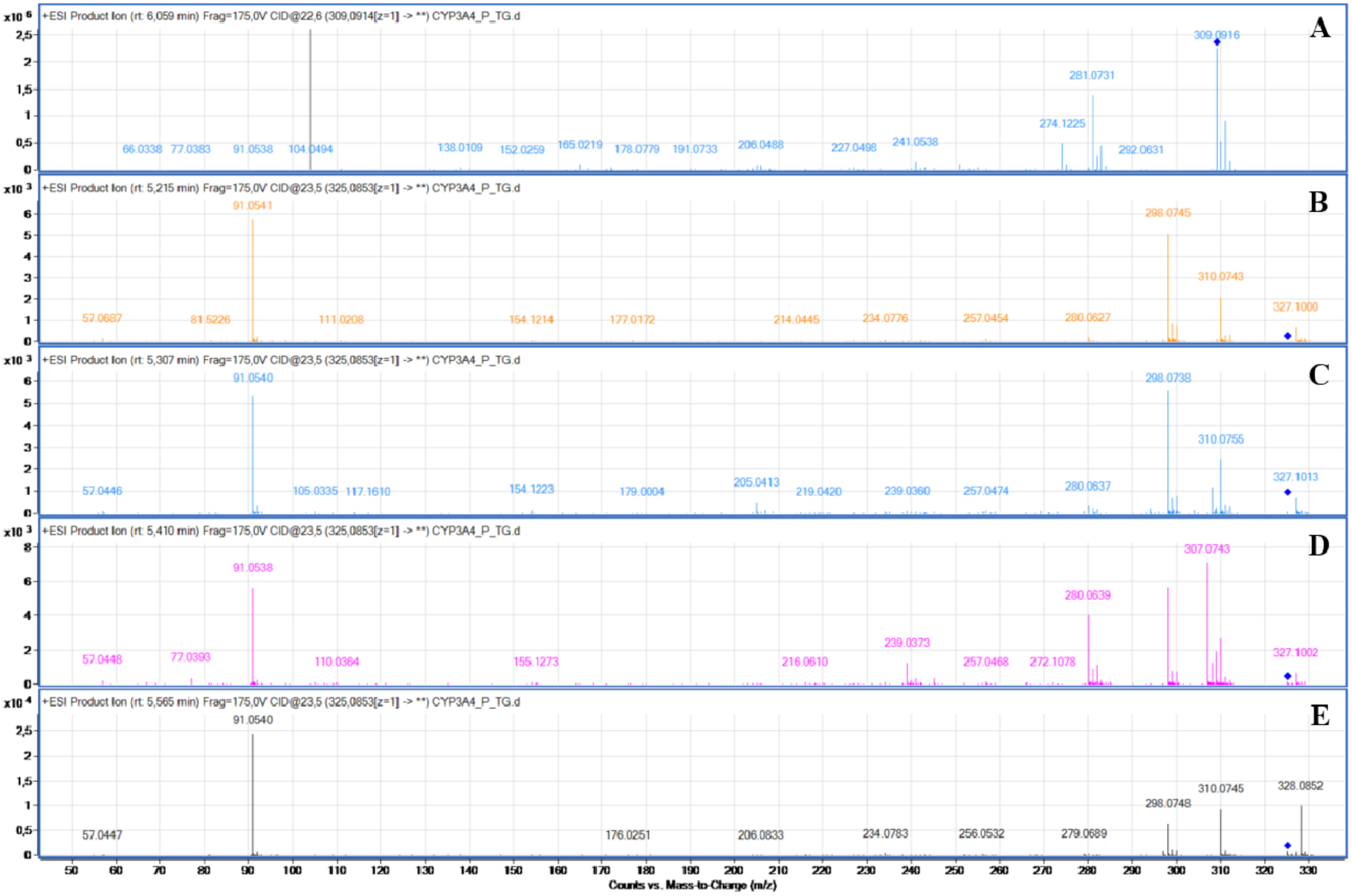

+ESI Product Ion (rt: 6,059 min) Frag=175,0V CID@22,6 (309,0914[z=1] -> **) CYP3A4_P_TG.d
A
x10 6
66.0338
77.0383
91.0538
104.0494
138.0109
152.0259
165.0219
178.0779
191.0733
206.0488
227.0498
241.0538
274.1225
281.0731
292.0631
309.0916
+ESI Product Ion (rt: 5,215 min) Frag=175,0V CID@23,5 (325,0853[z=1] -> **) CYP3A4_P_TG.d
B
x10 3
57.0687
81.5226
91.0541
111.0208
154.1214
177.0172
214.0445
234.0776
257.0454
280.0627
298.0745
310.0743
327.1000
+ESI Product Ion (rt: 5,307 min) Frag=175,0V CID@23,5 (325,0853[z=1] -> **) CYP3A4_P_TG.d
C
x10 3
57.0446
91.0540
105.0335
117.1610
154.1223
179.0004
205.0413
219.0420
239.0360
257.0474
280.0637
298.0738
310.0755
327.1013
+ESI Product Ion (rt: 5,410 min) Frag=175,0V CID@23,5 (325,0853[z=1] -> **) CYP3A4_P_TG.d
D
x10 3
57.0448
77.0393
91.0538
110.0364
155.1273
216.0610
239.0373
257.0468
272.1078
280.0639
307.0743
327.1002
+ESI Product Ion (rt: 5,565 min) Frag=175,0V CID@23,5 (325,0853[z=1] -> **) CYP3A4_P_TG.d
E
x10 4
57.0447
91.0540
176.0251
206.0833
234.0783
256.0532
279.0689
298.0748
310.0745
328.0852
Counts vs. Mass-to-Charge (m/z)

**Figure S2.** $MS^2$ spectra with different retention time of alprazolam and mono oxygen metabolites of alprazolam in the CYP3A biotransformated alprazolam sample. The first spectrum (**A**) represents alprazolam (precursor *m/z* 309.0902 ($[C_{17}H_{13}ClN_4+H]^+$), RT = 6.1 min, fragments of *m/z* 274, 281, and 292). The third (**C**) and fourth spectra (**D**) are the $MS^2$ spectra of two isomers of 4-hydroxy alprazolam (precursor *m/z* 325.0851 ($[C_{17}H_{13}ClN_4O+H]^+$), $RT_C$ = 5.3 min, $RT_D$ = 5.4 min, which are most similar showing fragment ions at *m/z* 310, 307, 298, 280, 257 and 239, especially the 307 (-$H_2O$), 280 (-NHCHOH) and 239 (-$C_3H_6N_2O$). The second $MS^2$ spectrum (**B**) is assigned to 5-N-O alprazolam (precursor *m/z* 325.0851 ($[C_{17}H_{13}ClN_4O+H]^+$), $RT_B$ = 5.2 min, closely eluting to the compounds at RT = 5.3 min/5.4 min) showing the common fragment ions at 310, 298, 280 and 257, and a new fragment with the MS at 214 (-$C_4N_3O$) and the absence of 307 can help to distinguish it from the spectra at 5.3 min/5.4 min. For the fifth spectrum (**E**, precursor *m/z* 325.0851 ($[C_{17}H_{13}ClN_4O+H]^+$), $RT_E$ = 5.6 min) the product ions at *m/z* 310, 298, 279, 256 and 206 represent the MS2 spectrum of α-hydroxy alprazolam.

**Table S1.** Sites of Metabolism (SoM) prediction for alprazolam with CYP3A4 on SMARTCyp.

| Atom identifier | Energy estimate [kcal/mol] | Corresponding metabolite |
|---|---|---|
| C.4 | 48.5 | $M_1$/$M_2$ (4α/β hydroxy alprazolam) |
| C.α | 59.9 | $M_3$ (α-hydroxy alprazolam) |
| N.5 | 61.9 | $M_4$ (5N-O alprazolam) |
